# Commentary: Long-Term Anatomical and Functional Survival of Boston Type 1 Keratoprosthesis in Congenital Aniridia

**DOI:** 10.3389/fmed.2021.815926

**Published:** 2021-12-23

**Authors:** Yelin Yang, C. Maya Tong, Andrea Dahoud, Mona Harissi-Dagher

**Affiliations:** Department of Ophthalmology, University of Montreal, Montreal, QC, Canada

**Keywords:** keratoprosthesis, keratoprosthesis (KPro), keratoprosthesis implantation, aniridia, aniridia associated keratopathy

We read with interest the article by Dyer et al. describing long term visual outcomes after Boston Keratoprosthesis (Kpro) in patients with aniridia (1). Optimizing visual outcomes is especially challenging in aniridic patients due to their high rate of preoperative comorbidities and postoperative complications. Visual outcomes can be maximized by utilizing a stage-based management approach and by reserving KPro surgery for high-risk stage IV patients and stage V patients with aniridia associated keratopathy (2). The authors describe overall retention rate of 83.3% with mean follow up of 58.7 months, which is comparable to other larger long-term studies in aniridia [76.9% with up to 38 months of follow up (3), 87% with mean follow up of 54 months (4)]. While 6/12 (50%) of eyes showed improvement in visual acuity by a mean of 0.7 logMAR and maintained ≤ 1.3 logMAR, 6/12 (50%) eyes in this study had reduced vision >1.3 logMAR during follow up secondary to complications. These included retinal detachment, elevated intraocular pressure, endophthalmitis, and limited vision potential from ocular comorbidities (1). Aniridia has been found to be a significant risk factor for poor vision outcome (no light perception) in a Canadian study, with most causes from inoperable retinal detachment (5). Glaucoma is another major cause of vision loss postoperatively, due to difficulty with accurate measurement of intraocular pressure, progressive angle closure, and likely changed biomechanics following Kpro (6). As authors suggested, these special considerations in aniridia and higher rate of complications may warrant a separate subgroup classification.

Retroprosthetic membrane formation (RPM) remains the most common complication after Kpro in aniridia. This may be related to aniridia fibrosis syndrome, which involves progressive fibrosis from proximity of intraocular devices to immature vessels in the rudimentary iris (7). As intraocular lenses can lead to formation of membranes and act as scaffold for RPM, surgeons might consider having a low threshold to explant the intraocular lens and implant an aphakic KPro at the time of surgery (5). In addition, RPM has been linked to increased risk of retinal detachment (8). It would be interesting to compare the outcomes of patients with pseudophakic and aphakic Kpro in the study by Dyer et al., and whether there are differences in formation of RPM or retinal detachment.

3/12 (25%) eyes in this study were pediatric patients. This may have contributed to higher rates of postoperative complications, as KPro implantation in children is known to be associated with significantly higher rates of complications, device failure, and worse visual outcomes than might be seen in adults (9). Keratoprosthesis implantation is not generally recommended for pediatric patients at our center (9).

58% (7/12) of eyes had primary implantation of Kpro in this study (1). Due to the presence of limbal stem cell deficiency, penetrating keratoplasty (PK) alone carries a poor prognosis (10). Keratolimbal allograft can be performed prior to PK, but requires systemic suppression. For these reasons, we consider Kpro as a primary procedure in patients with aniridia. In a previous Canadian study from our center involving 26 eyes with aniridia, there was a trend toward better final BCVA with primary Kpro compared to secondary Kpro (3).

Lastly, progressive vision loss can occur over time due to increased postoperative complications (4). Thus, careful long-term monitoring with a collaborative team involving glaucoma and retina specialists is critical in these patients.

## Author Contributions

YY, CMT, and AD drafted the manuscript. MH-D edited the manuscript. All authors contributed to the article and approved the submitted version.

## Funding

This work was supported by University of Montreal FROUM research fund.

## Conflict of Interest

MH-D and CMT are advisory board members for Dompe. The remaining authors declare that the research was conducted in the absence of any commercial or financial relationships that could be construed as a potential conflict of interest.

## Publisher's Note

All claims expressed in this article are solely those of the authors and do not necessarily represent those of their affiliated organizations, or those of the publisher, the editors and the reviewers. Any product that may be evaluated in this article, or claim that may be made by its manufacturer, is not guaranteed or endorsed by the publisher.
